# Increased Risk of Systemic Lupus Erythematosus in Patients with Chronic Urticaria: A Systematic Review and Meta-analysis

**DOI:** 10.31138/mjr.34.2.121

**Published:** 2023-06-30

**Authors:** Palapun Waitayangkoon, Nipith Charoenngam, Thanaporn Ratchataswan, Ben Ponvilawan, Aunchalee Jaroenlapnopparat, Patompong Ungprasert

**Affiliations:** 1 Department of Medicine, MetroWest Medical Center, Tufts University School of Medicine, Framingham, MA, United States of America,; 2 Department of Medicine, Mount Auburn Hospital/Beth Israel Lahey Health, Harvard Medical School, Cambridge, MA, United States of America,; 3 Department of Pediatrics, John A. Burns School of Medicine, University of Hawaii, Honolulu, Hawaii, United States of America,; 4 Department of Internal Medicine, University of Missouri-Kansas City School of Medicine, Kansas City, Missouri, United States of America,; 5 Department of Rheumatic and Immunologic Diseases, Cleveland Clinic Foundation, Cleveland, Ohio, United States of America

**Keywords:** chronic urticaria, systemic lupus erythematosus, autoimmune diseases, association, meta-analysis

## Abstract

**Introduction:**

The association between systemic lupus erythematosus (SLE) and chronic urticaria (CU) has been suggested in the literature although the amount of evidence is still relatively limited. We aimed to combine all available studies on this association using systematic review and meta-analysis technique.

**Methods:**

Potentially eligible studies were identified from Medline and EMBASE from inception to February 2023 using search strategy that comprised of terms for “chronic urticaria” and “systemic lupus erythematosus”. The eligible study must consist of one group of patients with CU and another group of comparators without CU and must compare the prevalence of SLE in each group and report effect size with 95% confidence intervals (95% CIs). We extracted such data from each study to calculate a pooled odds ratio using the generic inverse variance method with random-effect model. Funnel plot was used to evaluate publication bias. Newcastle-Ottawa Scale was used to appraise the methodological quality of the included studies.

**Results:**

A total of 5,155 articles were identified. After two rounds of independent review by four investigators, five studies met the eligibility criteria and were included in the meta-analysis. The meta-analysis found an increased prevalence of SLE among patients with CU compared with individuals without CU with the pooled odds ratio of 5.03 (95% CI, 2.57–9.85, I2 of 93%).

**Conclusion:**

This systematic review and meta-analysis found that patients with CU had a significantly increased risk of SLE compared to individuals without CU.

## INTRODUCTION

Systemic lupus erythematosus (SLE) is a common autoimmune disease with a wide range of manifestations.^1^ Multiple genetic and environmental factors are implicated in the disease occurrence and progression, resulting in difference in severity and outcome of the disease.^2^ Skin is one of the most commonly affected organs. In fact, some of the cutaneous manifestations, such as malar rash, discoid rash and photosensitive dermatitis, are parts of classification criteria. Other relatively common skin findings include non-scarring alopecia, oral ulcers, purpura, and urticaria.^3–4^

Chronic urticaria (CU) is defined as the presence of urticaria for more than 6 weeks. When exogenous trigger can be identified, it is called chronic inducible urticarias (CIndU). However, such trigger is not identifiable in the majority of patients (up to 90%) and, thus, is called chronic spontaneous urticaria (CSU). Although the pathogenesis of CSU is not fully understood, autoinflammation is thought to play a pivotal role.^5^

An association between CU and some autoimmune conditions, such as autoimmune thyroid diseases, type I diabetes, rheumatoid arthritis, and celiac disease has been reported.^6^ The relationship between CU and SLE has been reported as well although the number of studies is still relatively limited. In this review, we aimed to combine all available current studies and investigate the association between CU and SLE using systematic review and meta-analysis technique.

## METHODS

### Design

This systematic review and meta-analysis was carried out following the recommendations of the Preferred Reporting Items for Systematic reviews and Meta-Analyses (PRISMA) statement.

### Search strategy

Potentially eligible studies were identified from publications indexed in Medline and Embase from inception to February 2023, which was independently conducted by two investigators (PW, NC). Search terms were derived from terms related to “Chronic urticaria” and “Systemic lupus erythematous”. Detailed search strategy is provided in Supplemental Material 1. No language limitation was applied.

### Study selection criteria

The eligible study must be cohort study that consisted of one cohort of patients with CU and another cohort of comparators without CU. The study must compare prevalence of SLE in each group and report effect size with 95% confidence intervals (95% CIs). Four investigators (PW, NC, BP, AJ) independently reviewed the eligibility of the retrieved articles. Different opinions were resolved by discussion with the senior investigator (PU). If two studies utilized the same database, only the study with the largest number of participants would be included.

### Data extraction

We used a standardized collection form for data extraction: last name of the first author, country of the study, study design, publication year, number of participants, recruitment of participants, diagnosis of CU, diagnosis of SLE, mean age of participants, percentage of female participants, comorbidities, and variable adjusted in multivariate analysis.

### Quality assessment

Two investigators (PW and NC) independently evaluated the quality of each study using the Newcastle-Ottawa quality assessment scale.^7^ Different opinions were resolved by discussion with the senior investigator (PU).

### Statistical analysis

Statistical analysis was performed using Review Manager 5.3 software from the Cochrane Collaboration. Point estimates with standard errors were retrieved from each study and were combined using the generic inverse variance method as described by DerSimonian and Laird.^8^ Random-effect model, instead of fixed-effect model, was used as the included studies had different background populations and methodology/protocols. The Cochran’s Q test was used to determine statistical heterogeneity. This statistic was further adjunct with the I^2^ statistic which quantifies the proportion of the total variation across studies that is from heterogeneity rather than coincidence. A value of I2 of 0 – 25% represents insignificant heterogeneity, 26–50% represents low heterogeneity, 51–75% represents moderate heterogeneity and >75% represents high heterogeneity.^9^ Funnel plot was used to investigate for the presence of publication bias.

### Statement of Human and Animal Rights

This article does not contain any studies with human or animal subjects.

## RESULTS

**Figure 1.**
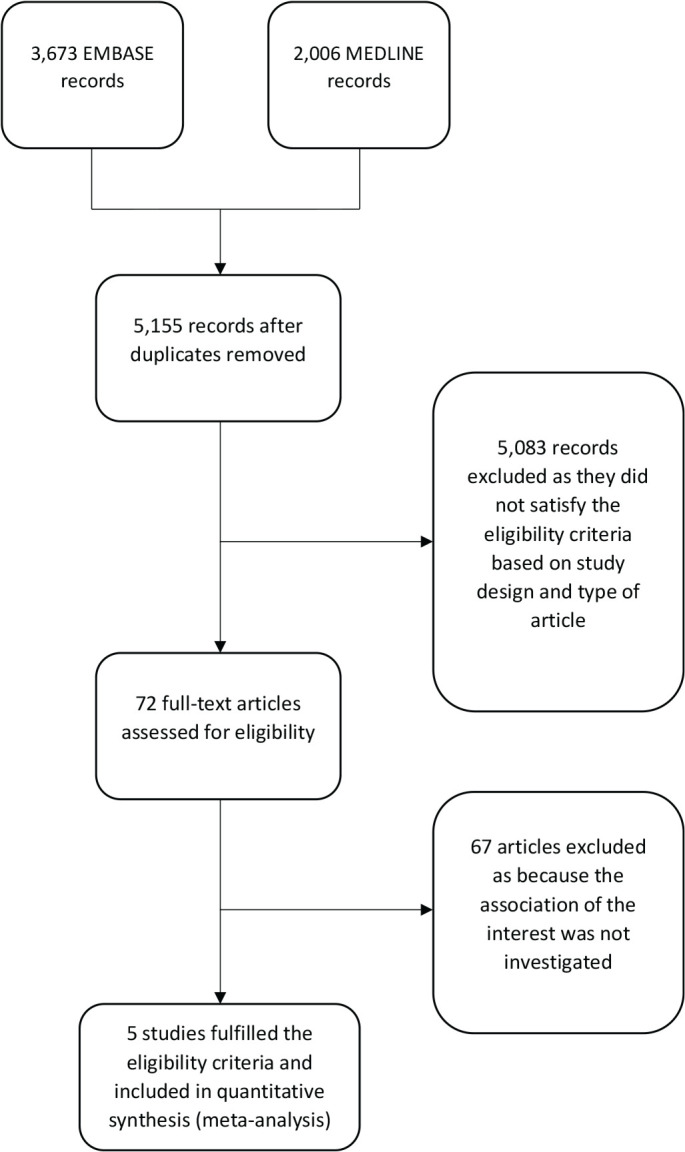
Study identification and literature review process.

We identified 5,679 articles from EMBASE and MEDLINE database, in which 524 were duplication articles, leaving 5,155 articles for title and abstract review. Of these, 5,083 articles were excluded as they did not satisfy the eligibility criteria based on study design and type of article. The remaining 72 articles were considered of interest and their full articles were retrieved for detailed evaluation. After full-text review, 67 articles were excluded as the outcome of interest was not reported, leaving 5 cohort articles for the meta-analysis.^6,10–13^ Figure 1 demonstrates the search methodology and selection process of this study. The characteristics of the included cohort studies are summarised in **Table 1**.

**Table 1.** Main characteristics of the cohort studies included in the meta-analysis.

	Confini-Cohen et al.	Kim et al.	Chiu et al.		Ghazanfar et al.	Le et al.
**Country**	Israel	Korea	Taiwan		Denmark	Canada
**Study design**	Retrospective cohort	Retrospective cohort	Retrospective cohort		Retrospective cohort	Prospective cohort
**Year of publication**	2012	2017	2018		2020	2021
**Patient/population (Patients with CU)**	Patients with CU were identified from Maccabi Healthcare Services (MHS) between January 1, 1993 and March 1, 2010. MHS is a 2-million-enrollee health maintenance organization, which contains information on all diagnoses and laboratory examinations that are performed at a single laboratory in central Israel.	Patients with CU were identified from Korean Health Insurance Review and Assessment Service – National Patient Sample (HIRA-NPS) database from January 2010 to December 2013. This is a national health insurance program that covers all citizens.	Patients with CU were identified from Taiwan Longitudinal Health Insurance Database (LHID) 2000, which is a subset of the National Health Insurance Research Database (NHIRD). NHIRD data are compiled by the Taiwan National Health Insurance (NHI) system which covered up to 99% of residents of Taiwan.		Patients with CU were identified from Danish National Patient Registry (DNPR) between January 1, 1994 until December 31, 2015. This registry contains health information of all citizens.	Patients with CU were prospectively recruited from Montreal Children’s Hospital Allergy and Immunology clinic from 2013 to 2019.
**Total number of patients with CU**	12,778	174,727	9,332		12,185	191
**Exposure**	Patients were diagnosed with CU by the presence of diagnostic codes of CU based on ICD-9-CM by either allergy and clinical immunology or dermatology specialists.	Patients were diagnosed with CU by the presence of diagnostic codes of CU based on ICD-10-CM plus history of antihistamine prescriptions for more than 6 weeks or antihistamine prescriptions in combination with one or more second prescription medications to treat CU	Patients were diagnosed with CU by the presence of diagnostic codes of CU based on ICD-9-CM		Patients were diagnosed with CU by the presence of diagnostic codes of CU based on ICD-8-CM until 1994 and ICD-10-CM from 1995 (diagnosed in specialised dermatological and allergology departments)	Patients were diagnosed with CU based on medical history and physical examination
**Comparators**	Comparators were patients who visited dermatologists, family physicians, or allergy specialists during the same period and were not given a diagnosis of CU. They were frequency matched with CU patients on the bases of age and sex.	Comparators without CU were randomly selected from the same database, using a randomised sampling method, stratified by age and sex.		Comparators without CU were randomly selected from the same database at a 1:4 ratio. They were matched to cases by number of dermatologic outpatient visits, sex, and age. Subjects with ambiguous or implausible basic data, such as conflicting entries for sex or date of birth, were excluded from the analysis.	Comparators without CU were randomly selected from the same database. They were matched on the bases of age and sex at a 1:10 ratio.	General paediatric population from a different study was used as comparator
**Total number of comparators**	10,714	5,487,073		37,328	104,007	852,190
**Outcome**	Presence of collected information on diagnostic history of SLE	Presence of diagnostic codes of SLE based on ICD- 10-CM		Presence of diagnostic codes of SLE based on ICD- 9-CM	Presence of diagnostic codes of SLE based on ICD-8-CM until 1994 and ICD-10-CM from 1995 on	Diagnosis of SLE reported by physician through a standardised questionnaire
**Average age of participants at index date (years)**	Patients with CU: 45.3 (18.5) Comparators: 44.2 (14.2)	Patients with CU: 42.82 (26.26) Comparators: 37.71 (20.8)		Patients with CU, mean (SD): 37.7 (17.6) Comparators, mean (SD): 37.7 (17.6)	Patients with CU: 38.4 Comparators: 38.8	Patients with CU (IQR) 9.4 (4.85, 13.65) Comparators: N/A
**Percentage of female**	Patients with CU: 66.3 Comparators: 85.	Patients with CU: 58.06 Comparators: 51.22		Patients with CU: 60.7 Comparators: 60.7	Patients with CU: 68.5 Comparators: 67.1	Patients with CU: 51.8 Comparators: N/A
**Variables adjusted in multivariate analysis**	None	Age and sex		Age, sex, and comorbidities	None	None
**Newcastle-Ottawa score**	Selection: 4 Comparability: 1 Outcome: 3	Selection: 4 Comparability: 1 Outcome: 3		Selection: 4 Comparability: 2 Outcome: 3	Selection: 4 Comparability: 1 Outcome: 3	Selection: 3 Comparability: 0 Outcome: 1

CU: chronic urticaria; ICD-8-CM: The International Classification of Disease, 8^th^ Revision, ICD-9-CM: The International Classification of Disease, 9^th^ Revision, Clinical Modification; ICD-10-CM: The International Classification of Disease, 10^th^ Revision; IQR: interquartile range; N/A: not available; SLE: systemic lupus erythematosus; SD: standard deviation.

In summary, among the five included studies, four of them including studies from Taiwan, Israel, Denmark, and Korea, were retrospective populational-based cohort. Only the study from Canada by Le et al. was prospective, single centred, and focused paediatrics. Most of the studies used diagnostic code for diagnosis of CU and SLE. All cohorts were cross sectional studies. Adjustment for the analysis of the association between CU and risk of SLE varied considerably across the included studies, ranging from none to extensive adjustment for demographic data and comorbidities. Most studies were of high quality as reflected by the high Newcastle-Ottawa score (**Table 1**).

### Association between CU and SLE

**Figure 2.**
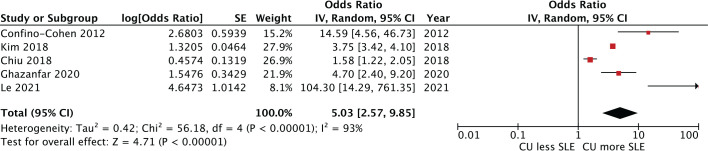
Forest plot of the meta-analysis of cohort studies.

**Figure 3.**
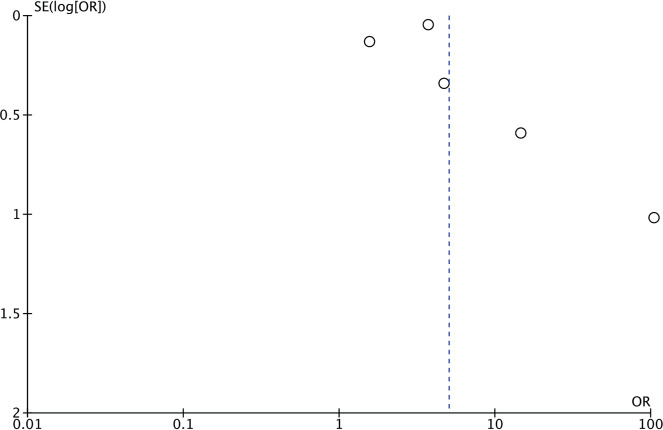
Funnel plot of the meta-analysis of cohort studies.

The meta-analysis found that patients with CU had a significantly higher risk of SLE with the pooled OR of 5.03 (95% CI, 2.57–9.85) with high statistical heterogeneity with I2 of 93% (**Figure 2**). Funnel plot of the meta-analysis of the studies was relatively asymmetric (**Figure 3**), indicating that publication bias may have been present.

## DISCUSSION

The current study is the first systematic review and meta-analysis that comprehensively investigates the association between CU and SLE. We found that patients with CU had approximately five-fold increased risk of SLE compared with individuals without CU. Although the exact underlying mechanism behind this observed association is not completely understood, there are some possible explanations.

The first possible explanation is shared immunopathogenesis as studies have revealed common immunologic markers in patients with CU and SLE. For example, patients with CU were shown to have increased levels of anti-dsDNA autoantibody, a specific marker of SLE, and anti-C1q autoantibody, a marker of severe SLE.^6,14–15^ In addition, patients with SLE have increased circulating IgG antibody to the α subunit of the high affinity IgE receptor (IgG anti-FcɛRIα), which is believed to play an important role in activation of mast cell in CU.^16^ It is therefore possible that presence of these autoantibodies seen in CU may trigger systemic autoimmunity of SLE in the same individuals later in their life. Second, CU and SLE may share an overlapping genetic risk as HLA-DRB1*04 was shown to be associated with both SLE and CU.^17–20^ Alteration in the gut bacteria may be another common risk factor for both CU and SLE^21^ as both diseases are associated with increased relative abundance of certain bacteria, such as Proteobacteria, Bacteroidetes and Actinobacteria.^22–24^ This notion is supported by the evidence that probiotics consumption in patients with SLE^21,25^ and CSU^26^ may mitigate clinical severity of the diseases by altering gut microbiota composition. Additionally, studies have shown that presence of *Helicobacter pylori* infection is another common risk of both CSU and SLE.^27^ It has been proposed that *H. pylori* triggers gut dysbiosis by producing the virulence factor cytotoxin-associated gene A and inducing hypochlorhydria and hypergastrinemia.^28^ There are some potential limitations in our study. First, some of the included studies did not provide a clear definition of CU, thereby jeopardizing the reliability of case identification. Second, the statistical heterogeneity of the meta-analysis was high (I^2^ 93%). This is likely due to different study design and participant characteristics. Third, publication bias may have been present. Additionally, most included studies did not adjust their outcomes and, therefore, the pooled results may be subjected to confounding effects.

The association of CU and SLE may have a clinical implication regarding screening for SLE as CU can be the first manifestation of SLE^29^ and may also suggest an unfavourable prognosis.^30^ Surveillance for other signs and symptoms of SLE as well as screening for autoantibodies could be helpful but further investigation is still needed.

## CONCLUSION

This systematic review and meta-analysis found that patients with CU had a significantly increased risk of SLE compared to individuals without CU.

## Figures and Tables

**Supplemental Material 1.** Searching strategy

**Ovid MEDLINE Database**1. rheumatic disease.mp. or exp Rheumatic Diseases/ 2. autoimmune disease.mp. or exp Autoimmune Diseases/ 3. systemic lupus erythematosus.mp. or exp Lupus Erythematosus, Systemic/ 4. sle.mp. 5. lupus.mp. 6. exp Urticaria/or urticaria.mp. 7. chronic urticaria.mp. or exp Chronic Urticaria/ 8. chronic spontaneous urticaria.mp. 9. acute urticaria.mp. 10. exp Vasculitis, Leukocytoclastic, Cutaneous/or urticarial vasculitis.mp. 11. dermatographic urticaria.mp. 12. cholinergic urticaria.mp. 13. cold urticaria.mp. 14. solar urticaria.mp. 15. idiopathic urticaria.mp. 16. aquagenic urticaria.mp. 17. 1 or 2 or 3 or 4 or 5 18. 6 or 7 or 8 or 9 or 10 or 11 or 12 or 13 or 14 or 15 or 16 **19.** 17 and 18
**EMBASE**1. ‘rheumatic disease’/exp OR ‘rheumatic disease’ 2. ‘lupus’/exp OR ‘lupus’ 3. ‘systemic lupus erythematosus’/exp OR ‘systemic lupus erythematosus’ 4. ‘lupus erythematosus’/exp OR ‘lupus erythematosus’ 5. ‘sle’/exp OR ‘sle’ 6. ‘urticaria’/exp OR ‘urticaria’ 7. ‘chronic urticaria’/exp OR ‘chronic urticaria’ 8. ‘acute urticaria’/exp OR ‘acute urticaria’ 9. ‘cholinergic urticaria’/exp OR ‘cholinergic urticaria’ 10. ‘cold urticaria’/exp OR ‘cold urticaria’ 11. ‘solar urticaria’/exp OR ‘solar urticaria’ 12. ‘aquagenic urticaria’/exp OR ‘aquagenic urticaria’ 13. ‘chronic spontaneous urticaria’/exp OR ‘chronic spontaneous urticaria’ 14. ‘urticarial vasculitis’/exp OR ‘urticarial vasculitis’ 15. ‘hives’/exp OR ‘hives’ 16. Hive 17. 1 OR 2 OR 3 OR 4 OR 5 18. 6 OR 7 OR 8 OR 9 OR 10 OR 11 OR 12 OR 13 OR 14 OR 15 OR 16 19. 17 AND 18
